# A Deep Learning Approach for Surface Crack Classification and Segmentation in Unmanned Aerial Vehicle Assisted Infrastructure Inspections

**DOI:** 10.3390/s24061936

**Published:** 2024-03-18

**Authors:** Shamendra Egodawela, Amirali Khodadadian Gostar, H. A. D. Samith Buddika, A. J. Dammika, Nalin Harischandra, Satheeskumar Navaratnam, Mojtaba Mahmoodian

**Affiliations:** 1 School of Engineering, RMIT University, 124 La Trobe St, Melbourne, VIC 3000, Australia; samarakoon.egodawela@student.rmit.edu.au (S.E.); sathees.nava@rmit.edu.au (S.N.); mojtaba.mahmoodian@rmit.edu.au (M.M.); 2 Faculty of Engineering, University of Peradeniya, Peradeniya 20400, Sri Lanka; samithbuddika@eng.pdn.ac.lk (H.A.D.S.B.); dammikaaj@pdn.ac.lk (A.J.D.) nalin@ee.pdn.ac.lk (N.H.)

**Keywords:** concrete cracks, unmanned aerial vehicles (UAVs), deep learning, convolutional neural network (CNN)

## Abstract

Surface crack detection is an integral part of infrastructure health surveys. This work presents a transformative shift towards rapid and reliable data collection capabilities, dramatically reducing the time spent on inspecting infrastructures. Two unmanned aerial vehicles (UAVs) were deployed, enabling the capturing of images simultaneously for efficient coverage of the structure. The suggested drone hardware is especially suitable for the inspection of infrastructure with confined spaces that UAVs with a broader footprint are incapable of accessing due to a lack of safe access or positioning data. The collected image data were analyzed using a binary classification convolutional neural network (CNN), effectively filtering out images containing cracks. A comparison of state-of-the-art CNN architectures against a novel CNN layout “CrackClassCNN” was investigated to obtain the optimal layout for classification. A Segment Anything Model (SAM) was employed to segment defect areas, and its performance was benchmarked against manually annotated images. The suggested “CrackClassCNN” achieved an accuracy rate of 95.02%, and the SAM segmentation process yielded a mean Intersection over Union (IoU) score of 0.778 and an F1 score of 0.735. It was concluded that the selected UAV platform, the communication network, and the suggested processing techniques were highly effective in surface crack detection.

## 1. Introduction

Cracks in structures can be detrimental to the longevity of civil structures. Crack inspections are a vital part of infrastructure health surveys in construction and quality control for identifying structural weaknesses early and planning necessary maintenance [1]. Visual inspection of cracks still remains the primary detection method and is most commonplace in the industry. However, effective manual inspection is infeasible in hard-to-reach superstructures such as high-rise buildings, tanks, pipelines, and space structures. Moreover, the subjective judgment of the inspector plays a pivotal role in the detection process and could cause irregularities and non-uniformities in the inspection process [2]. Due to challenging operational demands, unmanned aerial vehicles (UAVs) have become the most convenient and at times the only option for safe and successful data collection. 

UAVs for inspecting structures have been at the forefront of civil engineering research in recent decades [3]. Sensors such as Ultrasonic Pulse Velocity (UPV) testing, Ground Penetrating Radar (GPR) testing, and Acoustic Emission (AE) housed in a probe end have been tested previously with reasonable success [4] for crack inspection. However, a probe end requires complex robotics to maneuver and precise positioning readings to work in a confined setting [5]. Therefore, using imaging sensors for crack inspection can be an effective approach, especially for confined indoor applications. Simple visual inspection via imaging hardware mounted on UAVs can be a convenient, cost-effective method for understanding the condition of inaccessible superstructures [6]. The automatic detection of cracks in image/video data is again a field of research that has had a meteoric rise in popularity due to advancements in imaging technologies. However, analyzing the image/video data can be tedious, time-consuming, and error-prone due to human intervention [3]. 

A comprehensive review of the literature reveals that many UAVs, as listed in Table 1, are predominantly designed for outdoor use and rely on GPS or GNSS for navigation. This reliance provides accurate localization but limits their applicability in environments with weak or absent localization signals. This limitation almost completely renders them unsuitable for close-quarter operation, where the UAV needs to navigate confined spaces for inspection, with positioning data often weak or completely unavailable. Additionally, the larger size of these UAVs restricts their maneuverability in confined spaces, making them less suited for detailed inspections in such settings.

**Figure 1:**
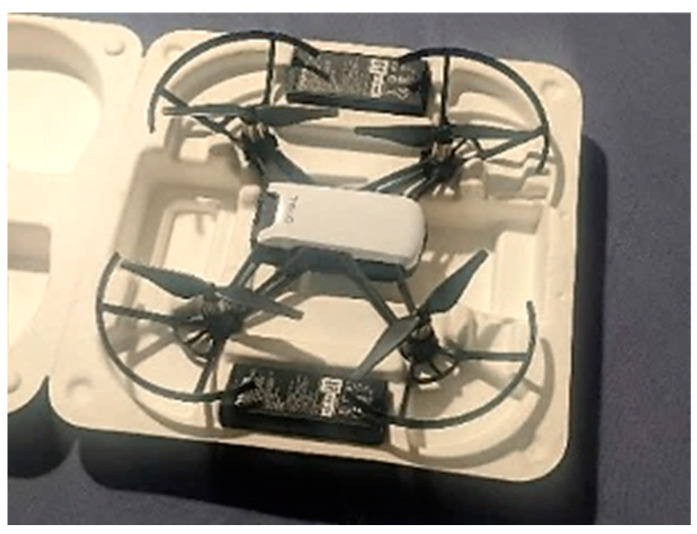
Ultra-lightweight UAV used in the study—DJI Ryze Tech Tello. The Tello is a compact UAV with small dimensions, designed to be easily maneuverable in confined spaces.

In contrast, this study utilizes the DJI Ryze Tech Tello (by Ryze Tech, Shenzhen, China) a commercially available, ultra-light UAV [11], to inspect surface cracks in structures. The UAV, shown in Figure 1, is particularly suitable for close-quarter applications due to its compact layout and agile maneuverability. The drone utilizes a VPS (Vision Positioning System) [11] equipped with a downwards-facing camera and an infrared sensor to stabilize itself and maintain its position and does not rely on GPS/GNSS signals. The UAV is suited for applications where accessing and exploring confined areas with limited access is required without the need for long-distance flights, i.e., indoor infrastructure, bridges, storage silos, etc. [12]. Table 2 shows the compact footprint of the UAV and its camera sensor information.

The accurate manual detection of cracks from the image/video data collected from a UAV can be tedious and time-consuming. Leveraging Artificial Intelligence (AI) and Machine Learning (ML) in combination with data captured through UAVs can significantly enhance the reliability and accuracy of inspections [13]. For this purpose, researchers have employed various image-detection methods, including morphological image processing [14], foreground–background separation [15], filtering [16], and percolation models [17]. However, these algorithms often face challenges in achieving robust generalization due to the interference of debris and environmental noise in practical engineering environments [14]. Moreover, most of these methods are highly sensitive to image scale, which is crucial when dealing with cracks of different scales and shapes [18]. Convolutional neural networks (CNNs), through feature learning, are invariant to change in the scale of images, making them highly adaptable to diverse and complex visual recognition tasks [18]. CNNs use object detector algorithms such as Single Shot Detector (SSD) [19], R-CNN and Faster R-CNN [20], and YOLOVx models [21]. Although the base CNN architectures embedded within object detectors generalize well in on-field settings, the region proposal networks used to produce box predictions generalize poorly due to being highly susceptible to overfitting to datasets [22].

Furthermore, object detectors can be vulnerable to adversarial attacks, where small, imperceptible perturbations in the input image can lead to incorrect object detections [23]. Object detectors produce box predictions and do not produce pixel-level granularity in crack detection, which is required for crack-width calculations [24]. Therefore, more recently, researchers have employed semantic image-segmentation algorithms to obtain pixel-level granularity in crack detection, such as Fully Convolutional Network (FCN) [19], UNet [25], and Recurrent Neural Network (RNN) [26]. These can be extremely resource-hungry and require powerful hardware, such as GPUs or TPUs that consume a substantial amount of energy. However, in the recent past, with the advent of large-scale pre-trained “foundation” models, there has been a shift towards transformers for visual recognition tasks [27]. With pre-trained transformer networks and lightweight decoders that run on edge computing hardware, multi-modal zero-shot inference in both natural language and images has become a reality. Some notable examples of such systems include Generative Pre-trained Transformers (GPTx) [28], the Language Model for Dialogue Applications (LaMDA) [29], Vision Transformer Detectron (ViTDet) [30], and the Segment Anything Model (SAM) [31]. Running only the decoder for inference, as opposed to passing the image through a deep CNN, not only improves efficiency but also enhances the model’s ability to perform well on a wide range of data, even in out-of-domain (OOD) applications [31]. The authors intend to suggest a multi-stage crack-detection approach in this paper. We use the power of CNNs to filter images with cracks without using typical object detection. Then, we combine this with a state-of-the-art segmentation method based on a transformer model called the Segment Anything Model.

This paper explores the feasibility of deploying two ultra-light UAVs and collecting image/video data of a structure with a classification system to filter out images that contain cracks, and an image-segmentation setup to identify pixels that correspond to cracks. The subsequent sections will delve into setting up a communication network for data collection through two assets of the UAVs, followed by an explanation of the processing algorithms for automatic crack identification and segmentation. First, a binary classification convolutional neural network (CNN) [32], designed to distinguish between images showing cracks and those without defects, is established. A novel CNN architecture will be introduced, named “CrackClassCNN”, which is tailored to detect cracks in images while maintaining minimal computational load. The proposed CNN architecture will undergo a comprehensive comparative analysis with state-of-the-art feature-extraction CNNs, and transfer learning will be employed for benchmarking [32]. After the initial stage of the identification of cracks though the CrackClassCNN, the transformer-based Segment Anything Model (SAM) will be employed to obtain pixel-level granularity of the cracks in the images. Additionally, an ablation study will be carried out to determine the most effective grid layout for sparse prompts, and the application-specific methodology for SAM prompting will be presented.

Key contributions of this paper are as follows:

- Testing the feasibility of a commercially available UAV for infrastructure inspection.
- Introducing a novel lightweight CNN architecture specifically tailored for infrastructure crack inspection named “CrackClassCNN”, and benchmarking its performance against existing transfer learning CNN architectures.
- Fine tuning the Segment Anything Model by suggesting a loss function and an effective prompting methodology for segmenting the pixels that contain cracks.

## 2. Methodology

**Figure 2:**
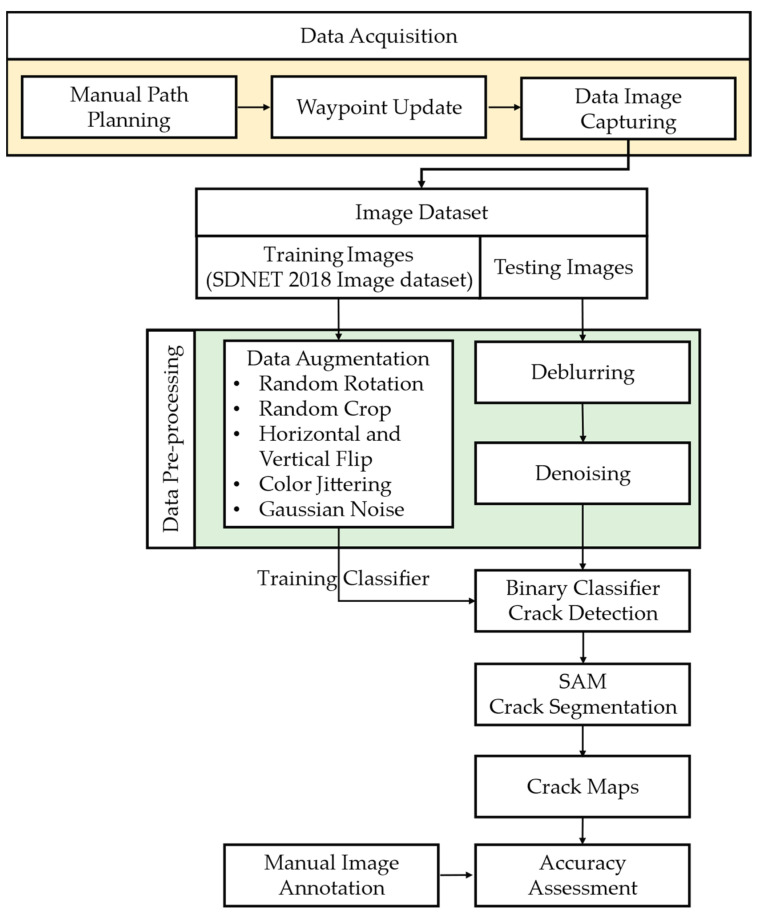
Methodology for data acquisition and the crack-detection process. This flowchart illustrates the step-by-step process for acquiring data and performing crack detection.

This section outlines the methodology for data collection and automatic processing to identify and segment structural cracks. The steps involved in the methodology are shown in Figure 2. Firstly, aerial images were obtained using two UAVs by conducting a mission along manually set-up waypoints to cover the complete region of interest. The captured image/video data were pre-processed to enhance their quality and clarity via a deblurring and denoising process. This is an important step for ensuring consistent results across image data and to improve the generalizability of the steps following suit. To further improve the domain generalizability of the models, a data augmentation step was introduced for the training set of the collected data. Subsequently, a crack-detection algorithm was employed to identify potential crack regions within the images. This algorithm utilizes a binary classification CNN to classify images into cracks and non-cracks. Transfer learning is utilized for comparing pre-trained CNNs, finetuned to binary classification, against the CNN layout introduced specifically for crack detection, named “CrackClassCNN”. This step replaces the need for an object detector which often can be prone to dataset overfitting issues, reducing their generalizability, as mentioned in the Introduction. Once the images that contain cracks are identified, a segmentation process is applied to obtain pixel-level granularity of the cracks within the images. For the segmentation task, a state-of-the-art image-segmentation model based on an autoencoder Vision Transformer model (ViT) by Meta research—the Segment Anything Model (SAM)—is used to provide a semi-supervised segmentation of the pixels with cracks. This is an important step as obtaining pixel-level granularity is a vital piece information for crack-width estimation during infrastructure inspection and health-monitoring tasks.

### 2.1. Data Acquisition via UAVs

This study was conducted on a structure at the Faculty of Engineering, University of Peradeniya (7.2537, 80.5916), Sri Lanka. The images captured were used to investigate and analyze the presence of cracks with the goal of assessing the severity and extent of the cracks, which can provide valuable insights into the structural integrity and maintenance requirements of the structure under observation. The complexity of the structure with confined spaces and manually inaccessible areas justified a UAV inspection process; thus, the UAVs served as practical tools to facilitate comprehensive data collection.

**Figure 3:**
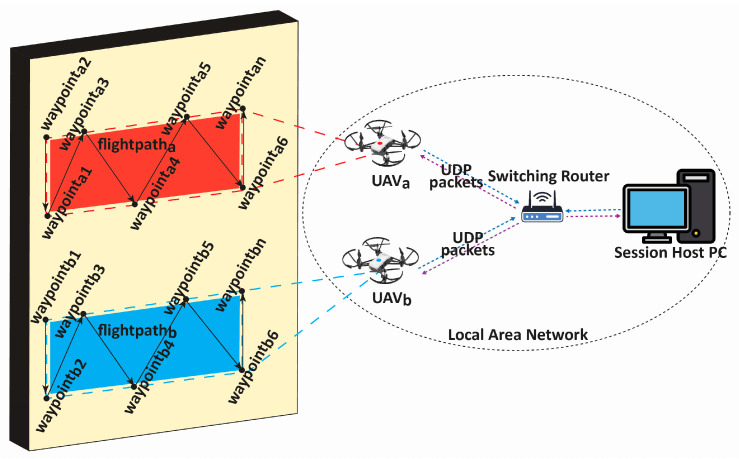
Communication network for data collection through UAVs. Shows the work involved in coordinating the movements and positions of the UAVs. User-defined waypoints and trajectories guided the drones during the mission, ensuring comprehensive coverage of the target structure.

In this work, two ultra-lightweight UAVs (Ryze Tech Tello by DJI) were employed to cover the structure of interest and collect image/video data. A robust and reliable communication network was set up for networking the UAVs with the session-hosting Personal Computer (PC). A set of pre-determined waypoints and flight paths were defined on the session-hosting PC, ensuring efficient coverage of the inspection area and avoiding redundant or overlapping scans. During the waypoint determination and path planning, factors such as the structure’s shape, potential obstacles, and any specific areas of interest that required closer inspection were considered. The distance from which a drone captures images is a critical factor in crack detection, as it directly impacts the visibility and resolution of crack coverage on the image. Special care was taken during path planning to maintain a consistent distance of 1.2 m from the wall during data collection. This deliberate planning allowed us to capture images from a fixed and controlled distance, minimizing field-of-view variations that could potentially affect the performance of ensuing models. Communication between the UAVs and the PC was managed through a Local Area Network (LAN) [11] using the User Datagram Protocol (UDP) [11], chosen for its efficiency and reliability in real-time applications. Here, an access point router with a unique Service Set Identifier (SSID) creates a LAN to which the UAVs and the hosting PC connect to. The host PC, which executes Python code, connects to the LAN Wi-Fi network to send and receive UDP commands via a predefined script containing the information of the waypoints and flight plans. The hosting PC executes the script by first establishing the UDP server and, after, sending control commands to the UAVs and listening for incoming data on the status of the drone (battery and Wi-Fi strength), as well as streaming image/video data. For this, the Tello SDK provided by Ryze robotics is used [33]. As shown in Figure 3, this network setup was instrumental in facilitating seamless data transmission, including the images and videos required for the study.

**Figure 4:**
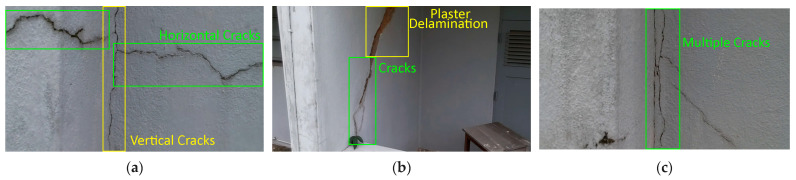

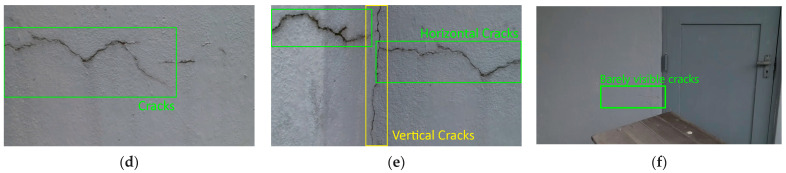
Image data collected of the structure under inspection using the UAVs. The Subfigures (**a**–**f**) shows the surface conditions with clearly visible cracks (**a**–**e**), exhibiting a combination of horizontal and vertical fractures, multiple cracks, and evident masonry plaster deterioration and barely visible defects (**f**).

A total of 150 images and 20 min of video data at 30 fps (~360,000 image frames) were collected, covering a selected building of the premises. Figure 3 shows some image data collected of the structure under inspection using the UAVs. As is evident from Figure 4, the collected images revealed the presence of cracks, structural deformations, corrosion, displacement, and the deterioration of building elements within the structure.

### 2.2. Pre-Processing Algorithms

The images captured by the UAV cameras are inevitably subject to the vibrations and perturbations of the platforms and motion. Furthermore, the imaging sensors are constantly moving during the exposure time of the camera. This introduced the blurring of images during the image-capturing process. Moreover, it was noticed that the communication between the UAV and the PC via the LAN channel introduced grainy speckle noise onto the image. To mitigate the effects, the obtained images were subjected to deblurring and denoising pre-processing steps to recover them to a workable level. Before the pre-processing steps, data augmentation techniques were applied to the data for greater generalizability.

#### 2.2.1. Data Augmentation

Data augmentation techniques play a pivotal role in the realm of deep learning, especially in computer vision tasks, by enhancing the diversity of training data and ultimately bolstering the generalization capabilities of models. The training set used for the training of binary classification CNNs (as explained in Section 2.3) was subjected to a data augmentation process via introducing random rotation, where images are rotated by arbitrary angles, replicating the natural variability in an object’s orientation with random values between −45 degrees and +45 degrees. Random cropping in the range of 50–90% was introduced, which extracted random sections of images of the said percentages, enabling the model to recognize different facets of an object and ensuring more generalizability for scale variability. The horizontal- and vertical-flipped mirrors images accommodated varying object orientations in the training dataset. Random zooming in and out in the range of 90–110% was introduced to randomly mimic shifts in perspective. Color jittering of a factor between 80 and 120% for brightness, contrast, saturation, and hue was performed to mirror diverse lighting scenarios. Lastly, the introduction of Gaussian noise infused images with real-world noise, contributing to the model’s resilience and adaptability to noisy input data. These data augmentation strategies collectively enriched the training dataset, and are used to equip deep learning models with better generalization and to help them excel in real-world scenarios.

#### 2.2.2. Deblurring

In this work, a state-of-the-art image deblurring technique suggested in MPRNet [34] was employed for deblurring. The method uses a multi-stage encoder–decoder setup to remove receptive field bottlenecks due to single-stage processes (such as in [35,36]). MPRNet avoids receptive field bottlenecks, preventing the downstream information loss associated with limited convolution kernel receptive fields. The repeated encoder–decoder architecture in MPRNet ensures larger receptive fields, preserving contextual information from preceding layers in the CNN.

**Figure 5:**
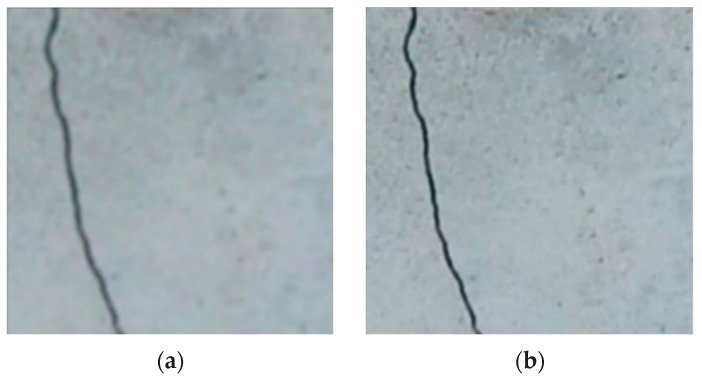
Comparison of original blurry image (**a**) versus deblurred image (**b**) achieved using the MPRNet CNN architecture. While the original image exhibits extreme blurriness, the deblurred image showcases remarkably sharp and well-defined crack edges.

Figure 5a shows the original image presented in this study, which suffers from a significant degree of blurriness, making it challenging to discern the cracks on the surface. However, through the application of the MPRNet CNN, the deblurred images shown in Figure 5b exhibit a remarkable transformation. MPRNet’s encoder–decoder setup significantly improves visual clarity, restoring fine details and accurately representing surface conditions while maintaining global features.

#### 2.2.3. Denoising

**Figure 6:**
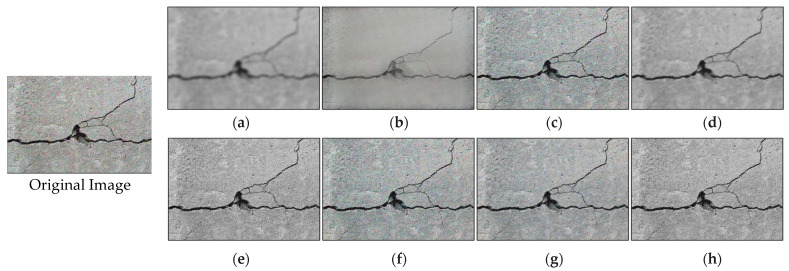
Visual representation of outputs of different denoising algorithms. (**a**) Gaussian denoising; (**b**) bilateral denoising; (**c**) wavelet denoising; (**d**) total variation denoising; (**e**) non-local means denoising; (**f**) shift invariant wavelet denoising; (**g**) anisotropic diffusion denoising; (**h**) block-matching denoising.

Image noise is an inevitable consequence of the thermal effects of imaging hardware and noise added due to interference in signal transmission [37]. The search for effective image denoising involves continuous trial and error due to the evolving nature of the field. To identify the most suitable denoising technique or combination, this study compared popular methods using Peak Signal-to-Noise Ratio (PSNR) (see Equation (1)), the Structural Similarity Index (SSIM) (see Equation (2)), and denoising time.

(1) $$\mathrm{PSNR}=10\cdot \log_{10}\left(\frac{{\left(L-1\right)}^{2}}{\mathrm{MSE}}\right)$$

where *L* = the maximum intensity level of the image; MSE = mean square error between the images.

(2) $$\mathrm{SSIM}\left(x,y\right)=\frac{\left(2\mu_{x}\mu_{y}+c_{1}\right)\left(2\sigma_{xy}+c_{2}\right)}{\left(\mu_{1}^{2}+\mu_{2}^{2}+c_{1}\right)\left(\sigma_{x}^{2}+\sigma_{y}^{2}+c_{2}\right)}$$

where $\mu_{x}$ = the average intensity of image *x*; $\mu_{x}$ = the average intensity of image *y*; $\sigma_{x}$= the variance of image *x*; $\sigma_{x}$ = the variance of image *x*; $\sigma_{xy}$ = the covariance of images *x* and *y*; $c_{1}={\left(k_{1}*L\right)}^{2};\;c_{2}={\left(k_{2}*L\right)}^{2};\;k_{1}=0.01;\;\mathrm{and}\;k_{2}=0$. In this study, denoising techniques, including Gaussian denoising, bilateral denoising, wavelet denoising, total variation denoising, non-local means denoising, shift invariant wavelet denoising, anisotropic diffusion, and block-matching denoising, were tested to enhance image clarity by reducing noise. Each technique tackled noise by addressing random variations and unwanted artifacts, ensuring the preservation of fine details, noise reduction, and maintaining the geometric integrity of the cracks during evaluation. Table 3 and Figure 6 present a comparative performance of these techniques. Total variation denoising had the highest PSNR (17.21) but exhibited high blurriness (Figure 6d) and a low SSIM value (0.60). Wavelet denoising struck a balance with high PSNR, high SSIM value, and reasonable denoising speed (approximately 1 Hz or 0.97 s execution time), making it the preferred choice. The denoised image using wavelet denoising is shown in Figure 6f.

### 2.3. Crack Detection

After the pre-processing steps, the images were passed through a convolutional neural network (CNN) for crack detection. CNNs are widely used for image classification tasks due to their ability to learn and extract meaningful features from images systematically. CNNs consist of multiple convolutional layers, followed by pooling layers and fully connected layers. Each convolutional layer applies a set of learnable filters to the input image, capturing different features at different levels of abstraction [32]. In this study, CNNs are used as a binary classifier to extract edge information corresponding to cracks and select the images that contain cracks.

**Figure 7:**
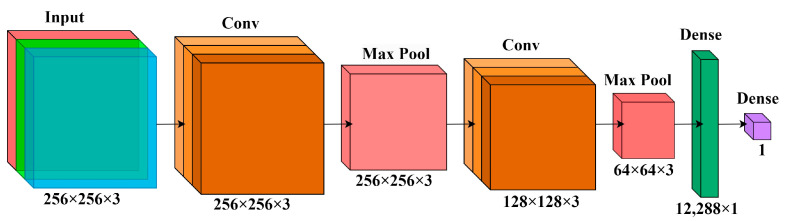
The CrackClassCNN architecture employs stacked convolutional layers for local feature extraction, followed by max-pooling layers to reduce dimensions. A densely connected layer culminates in a single-neuron output layer, optimizing speed and accuracy for crack detection.

The authors propose a novel CNN architecture named “CrackClassCNN” to perform the task of crack detection in images. After multiple implementations of differing CNN architectures, the layout in Figure 7 was chosen to be optimal for CrackClassCNN. Here, a trade-off between speed and accuracy was considered. Multiple iterations of training were run to test the layout with the fastest inference time and highest accuracy. Each layer in a CNN plays a specific role in feature extraction, spatial reduction, and prediction. Convolutional layers are the essential building blocks in CrackClassCNN. A convolutional layer’s main task is to detect an image’s local features. This is performed using 2D convolutional filters stacked parallelly. Hence, the convolutional layers learn features such as crack patterns that are useful in identifying recurring patterns in cracks from the images without cracks. After each convolutional layer, a pooling layer is deployed that is used to reduce the dimensions of the feature map generated by the convolutional layer. The max-pooling layer outputs the maximum value of the window in consideration. The window size and stride length are selected in such a way that the output dimensions of the image are scaled down by a factor of two after each convolutional layer. Finally, a dense layer consists of a single neuron connected to all the neurons of the previous layer, hence the term densely connected. The output layer is a dense layer consisting of a single neuron resulting in a single scalar value, which can be interpreted as the model’s prediction for a given input.

The accuracy of the suggested architecture for crack detection was benchmarked against existing feature-extraction CNN architectures by leveraging transfer learning. Pre-trained models employed in the previous literature, including Densenet201 [38], Xception [39], Mobile-netV2 [40], Resnet50 [41], and VGG19 [42], were used as the base feature-extraction CNN. The last layers of these pre-trained models were modified to suit the binary classification task by the addition of a flattened layer followed by two dense layers with ReLU activation. Sixty-four units were in the first dense layer and four units were in the second dense layer. This modification to the last layer of a CNN architecture is a standard approach for adapting a pre-trained model to a binary classification task. It enables the model to take the high-level features learned by the convolutional layers and make a binary classification decision.

This study used a portion of the SDNET2018 image dataset [43] as the training dataset for all the models mentioned. SDNET2018 is an annotated image dataset explicitly designed for training, validating, and benchmarking AI-based algorithms focused on concrete crack detection. This comprehensive dataset encompasses a total of 56,000 images representing various structures, such as bridge decks, walls, and roads. After an examination of the dataset, the ‘bridge deck’ section from SDNET2018 was eliminated, as we recognized that its distinct visual characteristics in terms of color and scale were not suitable for our application. This deliberate exclusion, coupled with a retraining effort incorporating hyperparameter optimization, resulted in a boost in classification accuracy. Notably, the integration of a data augmentation step proved instrumental in the network’s generalization capabilities. The construction of this pre-training dataset aimed to provide a diverse range of images, both with and without cracks, to facilitate the training of CNNs for concrete crack detection. For the validation of these models, the data collected in this experiment were used.

To ensure a fair comparison, all models, including the proposed architecture and the existing architectures, were trained using transfer learning for the same number of epochs (30 epochs). The training parameters were kept constant throughout the experiments, allowing a direct comparison of their performance on the given dataset and task. Deep CNNs are extremely taxing on hardware, as each image needs to be passed through the network once. It has been shown that increasing the depth of the convolutional network can increase the overall classification accuracy [32]. However, designing the network as light and shallow as possible is imperative to achieve real-time capabilities.

**Figure 8:**
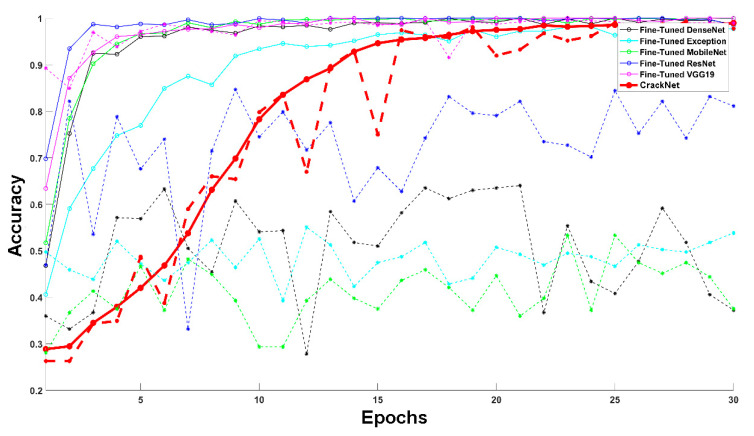
Variation in training (solid line) and validation accuracy (dashed line) of the different CNN architectures.

As can be seen in Table 4 and Figure 8, the CrackClassCNN architecture trained on SDNET2018 achieved an accuracy of 95.02% after 30 epochs of training, compared to other transfer learning architectures. Due to its lightweight design, the CrackClassCNN model offers a faster inference time of 0.55 s, compared to other models. Its streamlined architecture and efficient implementation allow for a quicker processing of images during the inference phase. This has practical benefits, especially in real-time or time-sensitive applications where prompt crack detection is crucial. Furthermore, the reduced computational requirements also make it feasible to deploy the model on resource-constrained devices, such as embedded systems or edge devices, without sacrificing performance.

**Figure 9:**
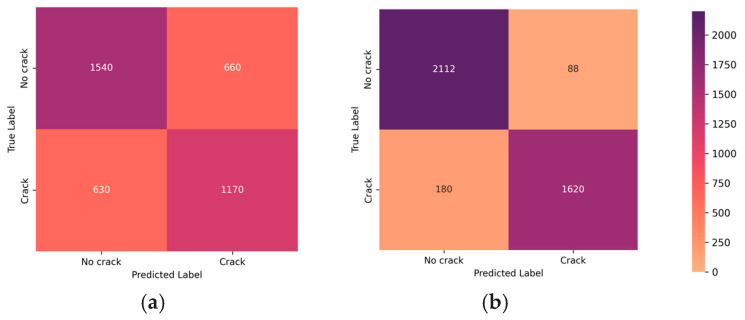
The confusion matrices before and after the denoising and blurring processing steps show the clear favorable bias of the classifier due to denoising. The figure showcases the classifier’s performance in distinguishing between positive and negative instances. The main diagonal contains true negatives where the classifier correctly identified the class. The transposed main diagonal contains the classifier’s incorrect predictions. (**a**) Before pre-processing; (**b**) after pre-processing.

In Figure 9, a comparative analysis of confusion matrices is presented, highlighting the impact of the denoising and blurring processing steps on the classifier’s performance. This visualization serves as a valuable insight into the classifier’s enhanced ability to distinguish between positive and negative instances after undergoing denoising, elucidating the positive influence of this processing step on overall classification accuracy.

**Figure 10:**
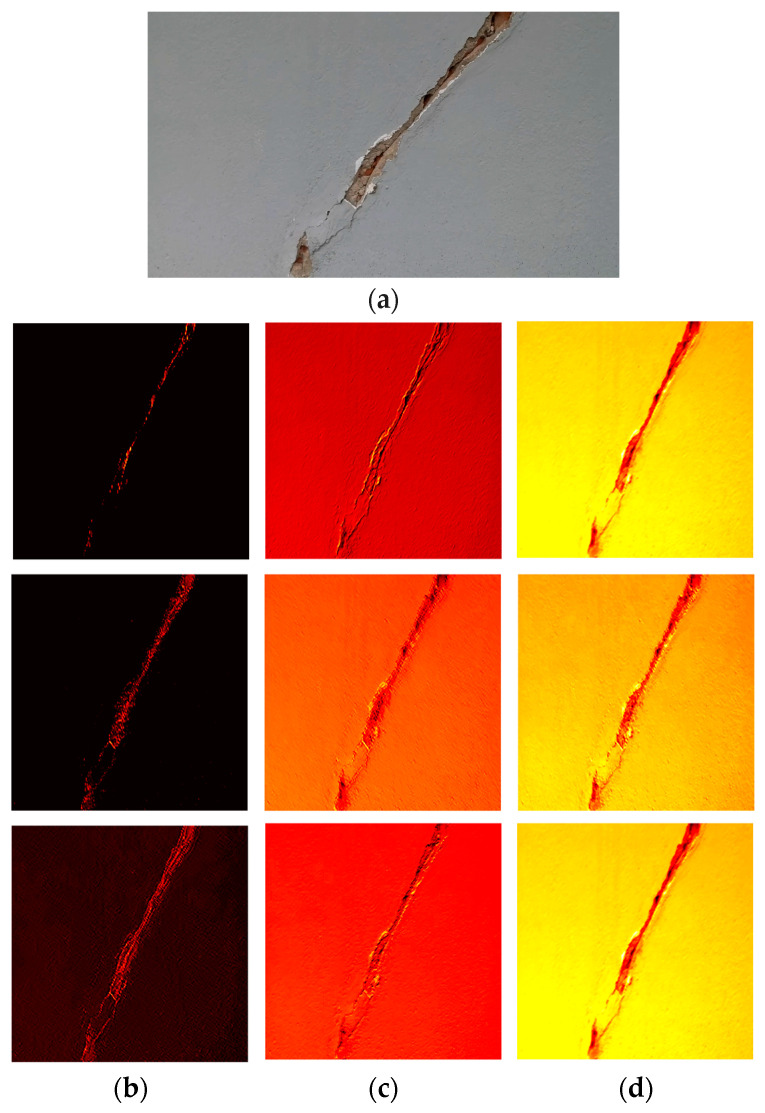
Visualization of feature maps of CrackClassCNN: The subfigures (**a**–**d**) illustrates the feature maps extracted from layers of the CNN. Each subfigure represents the output of a specific layer, showcasing the network’s ability to capture progressively abstract and complex patterns in the input image. The learned features in the subfigures show the hierarchical representation learned by the CNN.

Figure 10 shows the feature maps extracted at different layers of the CNN. The learned features showcased in the subfigures reveal the hierarchical representation acquired by the CNN. The evolving complexity of features showcases the CNN’s hierarchical learning process. From rudimentary details in early layers to sophisticated representations in later ones, this visualization underscores the network’s ability to discern and hierarchically represent diverse features present in the input data.

The trained CrackClassCNN classifier was tested on 4000 images from the data collected in the experiment. Figure 8 shows the effect of denoising and deblurring on the classifier’s performance. As is evident from the results, the wavelet denoising step chosen during the denoising stage and MPRNet deblurring affect the performance favorably regarding the binary classification of filtering images with cracks and rejecting the images without cracks for downstream image segmentation. These steps aim to counteract motion-induced blurring and mitigate the impact of speckle noise, ultimately enhancing the overall image quality. By deploying these pre-processing techniques, crack detection and segmentation operate on clear and sharp images, contributing to improved accuracy and reliability in diverse operational scenarios. This is apparent by both visual inspection and through the confusion matrix study, and it was decided that denoising and deblurring have a favorable impact towards the accuracy of the system.

### 2.4. Crack Segmentation Using Segment Anything Model (SAM)

After binary classification in the CNN, the images containing cracks are subjected to a segmentation process. The segmentation process aims to analyze and isolate the crack regions within these images [31]. The Segment Anything Model is an open-source segmentation algorithm developed by Meta AI, formerly Facebook, Inc. The SAM is designed to identify and segment various objects and regions within images. Its unique combination of automated segmentation and semi-supervised learning makes it a powerful tool for zero-shot transfer learning for pixel-level analysis and image-manipulation tasks. The SAM is trained on the largest segmentation dataset SA-1B, consisting of 1 billion image masks [31].

**Figure 11:**
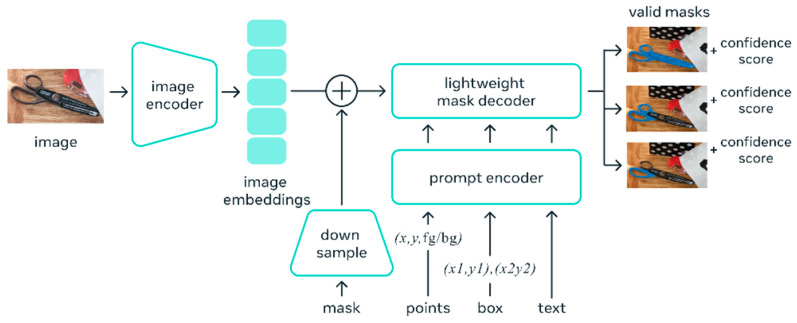
SAM layout, courtesy of Kirillov et al. [31].

The SAM (shown in Figure 11) utilizes the idea of “prompting”, which is an idea frequently used in Natural Language Processing (NLP) [44]. Given a sequence of words or tokens known as a prompt, the model is trained to predict the probability distribution of the possible next words or tokens, which is the basis of Natural Language Processing (NLP). In image segmentation, prompting could be a mask area, a set of points, a bounding box, or simply a text line provided to the model to give an idea of what it needs to segment in the input image. Figure 9 shows the high-level architecture of the SAM. The SAM works by encoding the prompt to a standardized representation called an embedding. All embeddings from different prompting methods are summed together at the element-wise summation, and a “complete embedding” is produced. Finally, the embeddings are sent through a modified transformer decoder block.

For segmenting the pixels that contain cracks, the authors use a sparse point grid of (nxn), covering the whole image to prompt the model. Given the prompt, the model results in multiple masks for the segmentation. The best segmentation mask depicting the cracked area was selected manually from the set of segmentation masks. By implementing the SAM, the authors expect to reduce the burden on a manual operator to select defective pixels by suggesting masks through the SAM. Furthermore, the SAM can run on web-based applications and can be accessed from any device with a web browser, allowing cross-platform compatibility.

#### 2.4.1. Fine-Tuning Segment Anything Model (SAM)

Fine-tuning the SAM involved training the edge mask decoder while leveraging a pre-trained foundation model as a checkpoint. This approach is crucial for efficiency, especially considering the resource-intensive nature of training the SAM, which necessitates 256 A100 GPUs and spans 3 to 5 days [31]. Training the entire model from scratch each time would be prohibitively expensive. The SAM offers a solution that allows users to load model checkpoints efficiently. This method can be beneficial for adapting the SAM to different datasets, resolutions, or architectures to enhance domain-specific accuracy. Initially, the foundation model checkpoint was loaded, utilizing the boilerplate code designed to be compatible with the Vision Transformer Base (ViT-B) architecture.

Selecting an effective loss function is critical in segmentation tasks, as it directly influences the model’s performance. The original SAM utilizes a combined loss function $CL\left(y,\hat{y}\right)\;$ (shown in Equation (3)), consisting of focal loss and dice loss, for semantic segmentation [31]. Dice loss $DL\left(y,\hat{y}\right)\;$ (given in Equation (4)) is used to solve the class imbalance issue in segmentation tasks; however, the use of dice loss can result in non-smooth optimization and a loss of gradient when the predicted or true positive regions have zero pixels [45]. Furthermore, focal loss $\;FL\left(y,\hat{y}\right)\;$ (given in Equation (5)) is highly sensitive to the appropriate choice of the focusing parameter γ [46]. The experimentation by the authors through the cross-validation of segmentation results with a parametric study for γ found the choice of γ led to sub-optimal results in terms of convergence and the accuracy of the results. Therefore, considering this, binary cross-entropy ($L_{bce}\left(y,\hat{y}\right)\;$, shown in Equation (6)), which is a simpler version of focal loss, was chosen to remove the effect of the focusing hyperparameter γ. Binary cross-entropy provides a smooth optimization landscape, reducing the likelihood of convergence issues during training. In this work, the class imbalance issue is solved through introducing class weight hyperparameter $w_{i}$. This is an important step in the fine-tuning process, as a large class imbalance issue was observed due to the pixels with cracks occupying only a small portion of the images and non-crack/background pixels being predominant in the dataset. Finally, an L2 regularization term containing the λ regularization strength and $\theta_{p}$ model parameters was introduced to the loss to prevent the overfitting of data. This term penalizes large individual weights (high $\theta_{p}$ values) in the model. The regularization strength λ controls the magnitude of this penalty. A sigmoid activation function was introduced after the binary cross-entropy for numerical stability. Regularization contributes to improved generalization by guiding the learning process toward simpler models that capture the underlying patterns in the data without being overly influenced by noise. The loss function ($L_{m-bce}\left(y,\hat{y}\right)$) used in this work is shown in Equation (7).

(3) $$FL\left(y,\hat{y}\right)=-\frac{1}{N}\overset{N}{\underset{i=1}{\sum }}{\left(1-\hat{y_{i}}\right)}^{\gamma }\;\cdot log\left(\hat{y_{i}}\right)\cdot y_{i}-{\hat{y_{i}}}^{\gamma }\;\cdot log\left(1-\hat{y_{i}}\right)\cdot \left(1-y_{i}\right)\;$$

(4) $$DL\left(y,\hat{y}\right)=\frac{1}{N}\overset{N}{\underset{i=1}{\sum }}1-\frac{2y_{i}\hat{y_{i}}+1}{y_{i}+\hat{y_{i}}+1}\;\;$$

(5) $$CL\left(y,\hat{y}\right)=\alpha FL\left(y,\hat{y}\right)-\left(1-\alpha \right)DL\left(y,\hat{y}\right)$$

(6) $$L_{bce}\left(y,\hat{y}\right)=-\frac{1}{N}\overset{N}{\underset{i=1}{\sum }}\left[y_{i}\cdot \log\left(\hat{y}\right)+\log\left(1-\hat{y}\right)\left(1-y_{i}\right)\right]$$

(7) $$L_{m-bce}\left(y,\hat{y}\right)=-\frac{1}{N}\overset{N}{\underset{i=1}{\sum }}w_{i}\left[y_{i}\cdot \log\left(\hat{y}\right)+\left(1-y_{i}\right)\cdot \log\left(1-\hat{y}\right)\right]+\lambda \underset{p}{\sum }\theta_{p}^{2}$$

where N is the number of samples or elements in the input, $y_{i}$ is the true binary label for the *i*th sample (either 0 or 1), $\hat{y_{i}}$ is the predicted probability for the *i*th sample in class 1, γ is the focusing parameter used in focal loss, λ is the regularization strength, and $\theta_{p}$ reflects the model parameters.

Hyperparameters were selected with a learning rate of 0.001, a batch size of 16, and a momentum term of 0.9 for stochastic gradient descent optimization. The transfer learning strategy involved using a pre-trained ViT-B architecture, adjusting the final classification layer, and freezing the early layers during fine-tuning. The training pipeline comprised 20 epochs, with a stepwise learning rate decay after every 5 epochs. Evaluation metrics such as Intersection over Union (IoU) and dice coefficient were used to assess segmentation performance. Visual examples, a few of which are shown in Table 5, revealed improved segmentation accuracy after fine-tuning, particularly in capturing smaller cracks and crack segments located at the edges of the crack tree. Future work should explore additional data modalities and address potential limitations in scenarios with complex backgrounds.

#### 2.4.2. Testing of SAM for Crack Image Segmentation

Fifty images were manually annotated with a training–testing–validation split of 80:10:10 for the fine-tuning of the SAM. The validation set of annotated images was used as a reference to compare against the segmentation results obtained from the SAM algorithm. Intersection over Union (IoU) (see Equation (8)) and F1 score (see Equation (9)) can be calculated by comparing the pixels in the segmented regions with the corresponding annotated regions in the ground truth. These metrics provide quantitative measures of how well the segmentation process aligns with the manually annotated regions. It is important to consider that manual annotation introduces subjectivity, and the accuracy of the segmentation process can be influenced by the quality and consistency of the manual annotations. Multiple annotators were used and consensus annotations were performed to help minimize potential bias or errors. Evaluating the accuracy of the segmentation process against the manually annotated images provides insights into the algorithm’s performance and facilitates necessary improvements.

**Table:** 

$IoU=\frac{Area\;of\;overlap}{Area\;of\;union}$	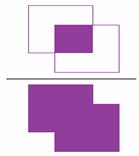	(8)
$F1\;score=\frac{2\times Area\;of\;overlap}{Total\;area}$	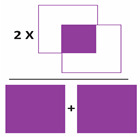	(9)

Data augmentation was performed on the training dataset to increase the diversity of the training set and help the model improve generalization to unseen data. As the pre-processing steps in place filtered out most of the blurriness and noise, these augmentation steps did not provide a considerable improvement to the results. However, improvements were observed for rotations of −25 degrees and +25 degrees, random cropping of 10–30%, color jittering of 50–90%, brightness of 90–110%, and a contrast of 90–110%. Table 6 shows some results of the SAM segmentation process.

#### 2.4.3. Ablation Study on the Number of Points (n) in Sparse Point Grid Prompting

As described earlier, the SAM requires a grid of point prompts with dimensions (n×n) for the segmentation. It is important to decide on the design parameter *n* as improper parameter designs will lead to imperfect segmentation with some crack paths being unprompted, and hence unsegmented by the SAM. This is especially pertinent when dealing with images featuring multiple targets of diverse sizes, as is often the case in images of cracks. Too sparse of a point grid may lead to certain crack patterns being unprompted and thus unsegmented, and raising the number of points in the point grid may lead to the division of a crack into multiple smaller fragments and result in a substantial increase in inference time. Consequently, there exists a trade-off between segmentation performance and testing efficiency. In this study, the authors tested *n* = 4, 8, 16, 32, and 64, and compared the testing efficiency in terms of IOU and F1 score against the inference time.

As can be seen in Table 7, the 32 × 32 grid layout yielded the most favorable balance between inference speed and inference accuracy. A clear leveling out in the accuracy figures can be observed as the point grid becomes denser. However, the adverse impact on the inference time is clearly observed.

## 3. Discussion

This work explored using two extremely lightweight UAVs to collect image/video data to detect surface cracks on structures. The study involved the collection of images of an indoor structure at the Faculty of Engineering, University of Peradeniya, Sri Lanka. The first step of data collection was to set up a communication network for the UAVs and the session-hosting PC. Following the setup, manual waypoints were determined to cover the structure effectively and efficiently. The collected image/video data were first subjected to deblurring and denoising processes. The pre-processed images were then sent through a binary classification CNN to filter out the images that contained cracks, removing the need for an object detector. The images filtered through the CNN were then sent through a transformer-based segmentation model (SAM) to obtain the pixel-level granularity of the cracks on the images. The SAM inference, which requires only a single pass through the decoder of the transformer, allows lightweight image segmentation. This is a departure from the resource-hungry image-segmentation algorithms in the literature.

The DJI Ryze Tello UAV, due to its compact footprint, is well suited for the inspection of space-deprived areas and especially suited for settings where GPS/GNSS navigation signals are not available. The use of two UAVs streamlines the image acquisition process and mitigates the inherent drawbacks of using an extremely lightweight UAV, such as limited range and flight time. However, it is observed that the manual flight planning for the coverage of the structure used in this work does not fully demonstrate the advantages of using multiple UAVs. Rather, to fully leverage the collective capabilities of multiple drones, a drone swarming methodology is recommended.

The core contribution of this work is the design of an automatic crack-detection and -segmentation methodology. It was observed that, during image capturing, the inherent motion of the UAVs introduced significant blur artifacts. The experimental results demonstrated by deblurring using MPRNet yielded significant improvements in image sharpness and detail restoration while preserving essential details. Following the deblurring process for the denoising task, wavelet denoising was observed as the best denoising technique among the tested methods to remove the image artifacts introduced due to the thermal effects of the imaging hardware and the noise added due to the interference in signal transmission. The pre-processed images were sent through a crack-detection CNN. A novel CNN architecture, CrackClassCNN, was proposed and compared against existing CNN models using transfer learning techniques from the literature. Herein, the objective was to evaluate the performance of the novel architecture in image classification tasks and determine its effectiveness compared to established CNN models. The experimental results demonstrated that the novel CNN architecture performed comparably to existing CNN models regarding classification accuracy and outperformed them in terms of lower inference time. Following crack detection using a CNN, the detected images were sent through a segmentation model (SAM). By leveraging the capabilities of the SAM, the authors observed a reduction in the need for manual intervention by providing suggested masks for identifying the defected regions. Furthermore, the number of target images for segmentation was significantly reduced by filtering the images that only contained cracks. This enables near real-time detection and increased reliability, and nods to the accuracy of the system.

The processing speeds were quantified at various stages. The data collection process through the UAVs can stream at approximately 15 Hz using the UDP server over the LAN to the central PC. A summary of the processing times for different stages of the workflow is as follows: the initial image processing, which includes deblurring and denoising, takes an average of 0.97 s per image; the binary classification through ‘CrackClassCNN’ takes 0.55 s per image on average; and the subsequent image segmentation using the SAM averages 0.79 s per image. These processing times were measured on a hosting device with an AMD Ryzen 4000 H-Series processor and an RTX 3060 6 GB GPU. This setup allows us to effectively process the data with a minimal delay, supporting the claim of near real-time detection. It is important to note that these timings represent the current capabilities of the system under the specified hardware conditions. Future enhancements in hardware and software algorithms may further reduce these processing times, moving closer to real-time analysis. For the purposes of this study, ‘near real-time’ refers to the capability of processing and analyzing data within a sufficiently short timeframe for practical on-site decision making in structural inspections.

*Failure Analysis of System*

It was deemed important to conduct an engineering failure analysis, as this provides crucial insights that enable improvements in design and future experiments. The UAV, though well-suited for inspecting confined spaces, faces limitations such as a short flight range (approximately 150 m) and a brief flight time (approximately 15 min), impacting the efficiency of infrastructure inspections. In such cases, the deployment of multiple UAVs in a collaborative manner becomes essential to overcome these constraints and ensure thorough inspection coverage. This approach allows for a more systematic and streamlined inspection process, leveraging the combined capabilities of multiple UAVs to cover larger distances and extend the overall inspection time [47].

**Figure 12:**
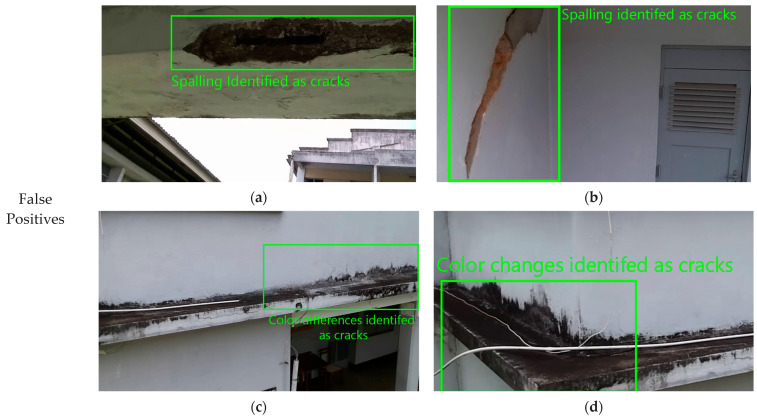

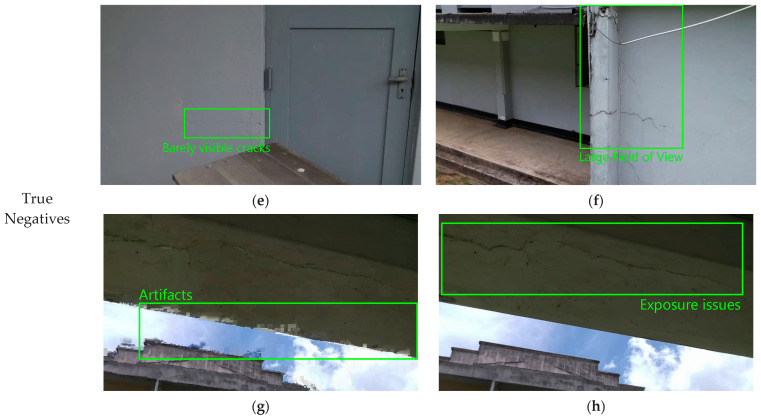
Classifier failure analysis. The image classifier encounters two main issues: false positives and false negatives. False positives include misidentifying non-existent cracks, especially confusing spalling with cracks due to similar appearances as shown in (**a**–**d**). False negatives occur when cracks, particularly barely visible ones, are missed as shown in (**e**). Scale relative to the camera’s view is critical, potentially causing smaller cracks to be overlooked (**f**). Extreme exposure issues causes both False positives and True negatives the cracks not being detected as shown in (**g**,**h**).

The image classifier has two main failure points: false positives and false negatives. The image classifier encounters challenges with false positives, misidentifying non-existent cracks and confusing spalling, especially, with cracks due to similar pixel appearances (Figure 12c,d). Extreme natural variations or surface irregularities may also be misconstrued as cracks, suggesting a need for a multi-class classification approach. False negatives arise when the system fails to detect cracks, especially barely visible ones (Figure 12e). The scale of cracks relative to the camera’s view is critical, with smaller cracks potentially being overlooked (Figure 12f). Furthermore, despite attempts through pre-processing to address image artifacts, cases arising due to packet loss or corruption (Figure 12g) can lead to missing or distorted parts of the image and can lead to undetected cracks. However, such extreme cases where the image is severely distorted occupy a small portion of the dataset (less than 2%), therefore resulting in the classifier performance of 95.02% accuracy for filtering cracks in an image.

The transformer-based SAM segmentation breaks an image into different segments or regions based on certain characteristics. However, if the field of view in the image is too wide, the segmentation latches on to too many local features to perform segmentation, resulting in a number of masks making it challenging to single out the crack mask (see Table 8). As a solution, the authors suggest fine-tuning the segmentation through a Human-in-the-Loop validation process to help ensure the accuracy of the masks and the overall performance of the crack-detection system. However, in all cases, the system can provide a fair estimation of the cracks in at least one of the masks, which can then be easily filtered manually by an operator in a semi-automated way, rather than the tedious manual selection of cracks in an image.

## 4. Conclusions

The UAV created by DJI, Ryze Tech Tello, is an ultra-lightweight and compact UAV well suited for inspecting cracks in confined indoor spaces where obstacles could be plentiful and positioning signals are often weak or completely unavailable. The core contribution of this work was to suggest a processing methodology that enables the automatic detection and segmentation of images with cracks. A novel CNN layout, CrackClassCNN, was network-trained on the dataset SDNET2018, excluding the bridge deck portion, reaching a classification accuracy of 95.02%. CrackClassCNN proved to be comparable with existing transfer-learning-based classifiers in terms classification accuracy, but with a faster inference time due to its simpler layout. Training the CNN’s data augmentation on the training dataset proved to be beneficial in improving classification results by improving the network’s generalizability. Following the filtering of images that contained cracks via the CrackClassCNN, the images were then sent through a transformer-based image-segmentation process using a fine-tuned Segment Anything Model (SAM). The fine-tuning process involved training the edge mask decoder by leveraging a pre-trained Vision Transformer Base (ViT-B) foundation model as a checkpoint. Here, a new loss function was introduced based on binary cross-entropy loss. Overall, the segmentation process resulted in good segmentation accuracy with an Intersection over Union (IoU) score of 0.851 and an F1 score of 0.728. Furthermore, the multi-stage processing methodology offers near real-time performance, ensuring the efficient and timely identification of cracks in the captured images.

It is important to note that the manual waypoint planning used in this work does not fully demonstrate the advantages of using multiple UAVs. To fully leverage the collective capabilities of multiple drones, future iterations of this work will incorporate more UAV assets and automated algorithms for dynamic path planning and obstacle avoidance, allowing for real-time adaptation to changing conditions, and optimizing the overall efficiency of the inspection process.

## Figures and Tables

**Table 1:** Recent work in crack detection using UAVs.

Study	UAV Platform	Image Sensor	Drone Dimensions (mm)	Weight (g)	Navigation Method
Kim et al. [7]	AiBotix X6	On-board sensor	1050 × 1050 × 450	4000	GPS
Dorafshan et al. [8]	DJI Mavic mini	On-board sensor	159 × 202 × 55	249	GPS
	DJI Inspire 1		438 × 451 × 301	3060	GPS
	DJI Phantom 4		360 × 280 × 170	1380	+GNSS
Kim et al. [3]	DJI Inspire 2	Zenmuse X5S image sensor	427 × 317 × 425	3440	GPS
Munawar et al. [9]	DJI-M200	On-board sensor	716 × 220 × 236	3800	GPS + GNSS
Cao et al. [10]	DJI Mini3 Pro	On-board sensor	191 × 245 × 62	249	GPS

**Table 2:** Details of the UAV and camera sensor [11].

Parameter	Value
Resolution	2592 × 1936
Weight	80 g
Dimensions	98 × 93 × 41 mm
Max Flight Distance	100 m
Max Flight Time	15 min
Lens Focal Length	80 g
Field of View	82.6°

**Table 3:** Denoising methods and their performance.

**Method**	**PSNR**	**SSIM**	**Time**
Original vs. Noisy Image	16.73	-	-
Gaussian Denoising	16.86	0.52	**0.04**
Bilateral Denoising	17.01	0.55	31.69
Wavelet Denoising	16.95	0.85	0.97
Total Variation Denoising	**17.21**	0.60	3.80
Non-Local Means Denoising	16.78	**0.88**	0.21
Shift Invariant wavelet Denoising	16.91	0.67	0.46
Anisotropic Diffusion	16.90	0.65	0.11
Block-matching and 3D filtering	16.96	0.77	4.08

Highlighted in bold are the best performing method for each performance metric PSNR, SSIM and Denoising Time.

**Table 4:** Relative performance of tested CNN architectures for the dataset.

Reference	Model	Testing Accuracy	Inference Time
Su et al., 2020 [38]	Fine-tuned Densenet_201	0.9345	2.25
Philip et al., 2023 [39]	Fine-tuned Xception	**0.9868**	2.01
Li et al., 2022 [40]	Fine-tuned Mobile-netV2	0.9398	1.56
Qayyum et al. 2023 [41]	Fine-tuned Resnet_50	0.9762	1.95
Ali et al., 2021 [42]	Fine-tuned VGG_19	0.9845	2.56
Ours	CrackClassCNN	0.9502	**0.55**

Highlighted in bold are the best performing model for each performance metric Testing Accuracy and Inference Time.

**Table 5:** SAM image-segmentation performance before and after fine-tuning.

Original Image	Before Fine-Tuning	After Fine-Tuning
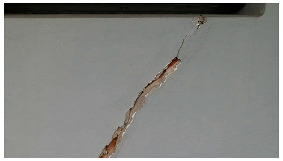	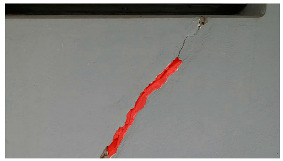	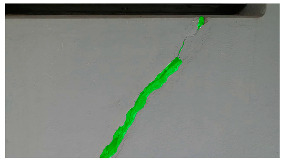
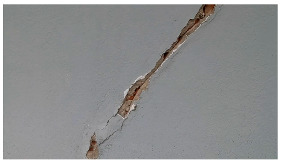	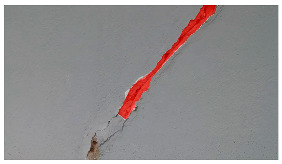	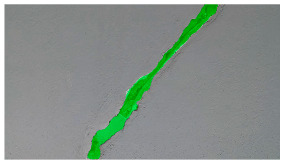
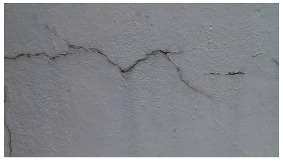	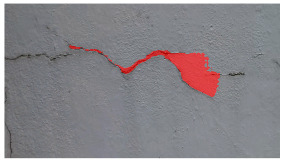	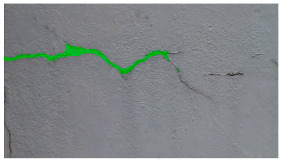
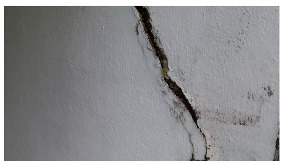	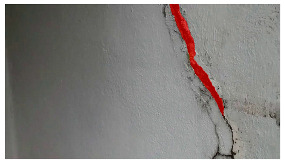	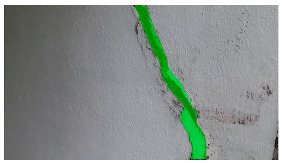

**Table 6:** Results of the segmentation process.

Original Image	Segmented Image	IOU	F1 Score
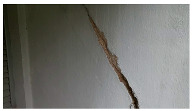	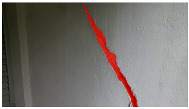	0.850	0.725
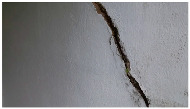	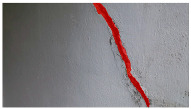	0.711	0.840
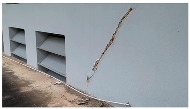	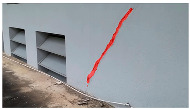	0.899	0.646
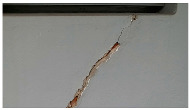	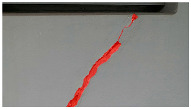	0.897	0.830

**Table 7:** Results of the ablation study.

Number of Points in Sparse Grid (n × n)	IOU	F1 Score	Inference Time (s/Image)
(4 × 4)	0.113	0.125	0.11
(8 × 8)	0.459	0.324	0.34
(16 × 16)	0.698	0.389	0.50
**(32 × 32)**	**0.851**	**0.728**	**0.79**
(64 × 64)	0.898	0.798	1.20

Highlighted in bold are the best performing grid layout for promting of the SAM model.

**Table 8:** Image segmentation resulting in multiple image masks shown in different colored masks.

Input Image	Mask Predictions
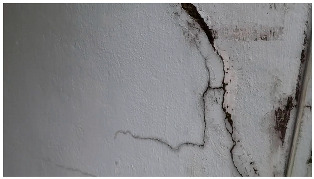	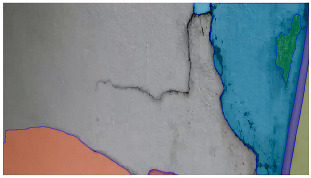
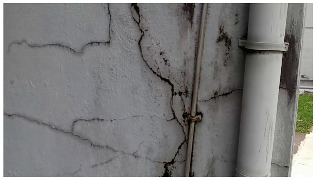	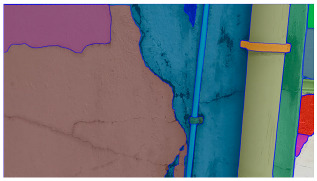
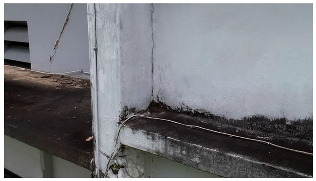	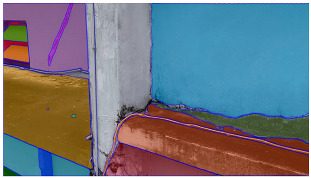

## Data Availability

The data collected in this study will be shared upon reasonable request.
